# Risk Stratification of Lamina and Pedicle Morphometry for Screw Fixation in the Axis Vertebra

**DOI:** 10.7759/cureus.104954

**Published:** 2026-03-09

**Authors:** Neha Xalxo, Laishram Sophia, Krishna S Patil, Shaival Shaival, Vandana Mehta, Lalit Ratanpara

**Affiliations:** 1 Anatomy, All India Institute of Medical Sciences, Rajkot, Rajkot, IND; 2 Anatomy, Employee State Insurance Corporation Medical College and Hospital, Faridabad, IND; 3 Anatomy, Balvir Singh Tomar Institute of Medical Sciences and Research, Jaipur, IND; 4 Orthopaedics and Trauma, Apollo Gleneagles Hospital, Kolkata, IND; 5 Anatomy, Vardhman Mahavir Medical College and Safdarjung Hospital, New Delhi, IND

**Keywords:** axis vertebra, morphometric study, pedicle screw, risk stratification, screw fixation, translaminar screw

## Abstract

Introduction: Screw fixation of the second cervical vertebra (C2), also known as the axis, is a mainstay in the management of upper cervical trauma and instability. The safety of C2 fixation via translaminar screw (TLS) and transpedicular screw (TPS) techniques is fundamentally influenced by individual anatomical variability. This study addresses the need to move beyond reliance on mean values by establishing quantifiable, integrated risk thresholds for both fixation pathways within the study population.

Methods: A total of 50 dry human C2 vertebrae were evaluated. The length, height, and thickness of the lamina were measured, and pedicle dimensions were recorded. Safety thresholds were defined according to commonly accepted minimum screw corridor requirements. Lamina and pedicle categories were cross-tabulated to determine the proportion of vertebrae that could safely accommodate laminar fixation, pedicular fixation, or both.

Results: The mean C2 laminar length and height demonstrated no statistically significant side-to-side differences (p>0.05), with a mean middle third laminar thickness of 5.8 mm bilaterally. Despite favorable mean values, laminar thickness decreased to 3.3 mm and pedicle width to 2.1 mm in some specimens, falling below accepted safety thresholds. Risk stratification of laminar thickness revealed that 36 vertebrae (72%) demonstrated bilateral laminar thickness ≥4.0 mm, indicating suitability for TLS fixation, while two specimens (4%) showed bilateral laminar thickness less than 4.0 mm, rendering them unsuitable for TLS fixation. This variability underscores the importance of individualized morphometric assessment prior to C2 screw fixation.

Conclusion: The quantified bilateral laminar risk identified in this study supports a paradigm shift in preoperative planning for C2 fixation. Translating morphometric measurements into a quantified safety model provides an objective framework to guide selection of the most appropriate fixation technique, thereby enhancing operative planning, reducing neurovascular risk, and ensuring selection of the safest available anatomical corridor for each patient.

## Introduction

The craniovertebral junction is a critical anatomical region that lodges crucial neurovascular structures [1] and allows maximum spinal mobility [2-4]. Instability in this region due to trauma, congenital anomalies, inflammatory conditions, or degenerative changes often necessitates surgical stabilization of the atlantoaxial complex.

The second cervical vertebra (C2), termed the axis, is an integral component of the craniovertebral junction and has an intricate anatomical relationship with the vertebral arteries [5,6]. In patients with polytrauma, spinal injuries are common, and nearly 55% of these involve the cervical spine [7]. Consequently, the axis is the most frequently fractured vertebra in upper cervical spine injuries [8]. Owing to its pivotal biomechanical role, secure fixation of C2 is essential for restoring stability and preventing neurological compromise.

Stabilization of the cervical spine following traumatic injury to this region is widely accomplished through translaminar screw (TLS) and transpedicular screw (TPS) fixation techniques. Inaccurate screw placement may result in significant injury to critical neurovascular structures, including the spinal cord, nerve roots, and vertebral arteries [9]. The narrow operative corridor and proximity to these critical structures render screw placement technically demanding and potentially hazardous, necessitating detailed anatomical studies of C2 relevant to neurosurgical procedures [10].

The safety of TLS and TPS fixation is primarily influenced by the morphometry of the lamina and pedicle. Reliance solely on mean values may obscure significant individual variability, especially in populations with smaller skeletal dimensions. This necessitates a shift from descriptive morphometric evaluation to quantifiable, risk-based safety stratification, which may help surgeons determine the safest fixation corridor specific to each patient. The present study was conducted on a sample of dry adult axis vertebrae from a North Indian population to facilitate safer, customized C2 screw fixation through detailed morphometric assessment of the lamina and pedicle.

## Materials and methods

This observational study was conducted in the Department of Anatomy, Vardhman Mahavir Medical College and Safdarjung Hospital, New Delhi, and 50 human dry adult axis vertebrae were examined. Vertebrae with gross deformity or degenerative changes were excluded from the study. The osteometric evaluation was carried out by using a digital vernier calliper (SKADIOO; Perfect Sales India, Haryana, India) with a sensitivity of 0.1 mm. 

Evaluation parameters

The following parameters of the lamina and pedicle of the axis vertebra were systematically evaluated.

Lamina Measurements

**Figure 1:**
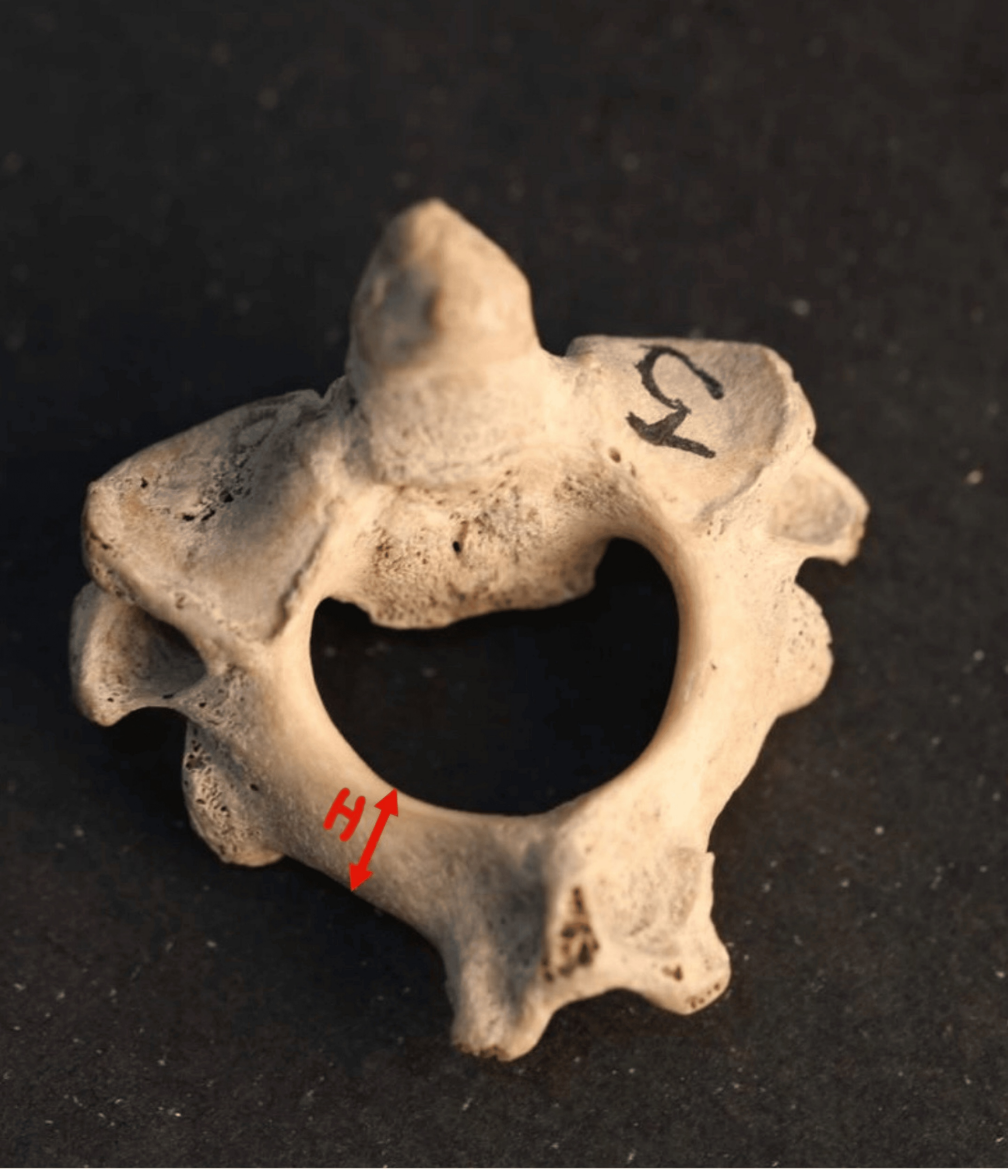
Measurement of the laminar height of the axis vertebra H = height of the lamina, measured as the distance between the upper and lower margins of the lamina. The axis vertebra was sourced from Vardhman Mahavir Medical College and Safdarjung Hospital.

Height: It was measured as the vertical distance extending from the upper margin to the lower margin of the lamina (Figure 1).

**Figure 2:**
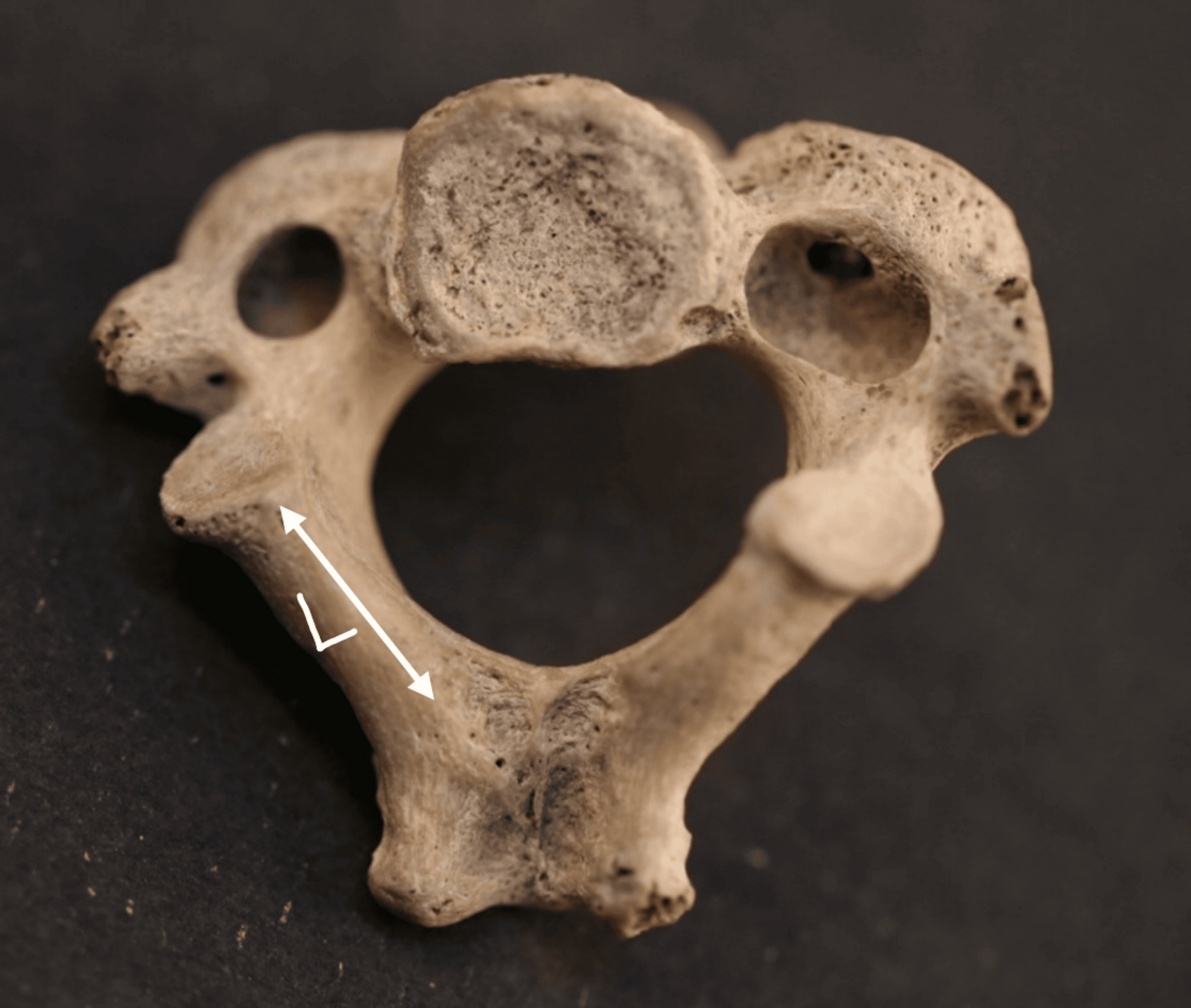
Measurement of the laminar length of the axis vertebra L = length of the lamina, measured as the distance from the posterior edge of the inferior articular facet (IAF) to the base of the spinous process. The axis vertebra was sourced from the Vardhman Mahavir Medical College and Safdarjung Hospital.

Length: It was determined as the linear distance from the posterior edge of the inferior articular facet (IAF) to the base of the spinous process (Figure 2).

**Figure 3:**
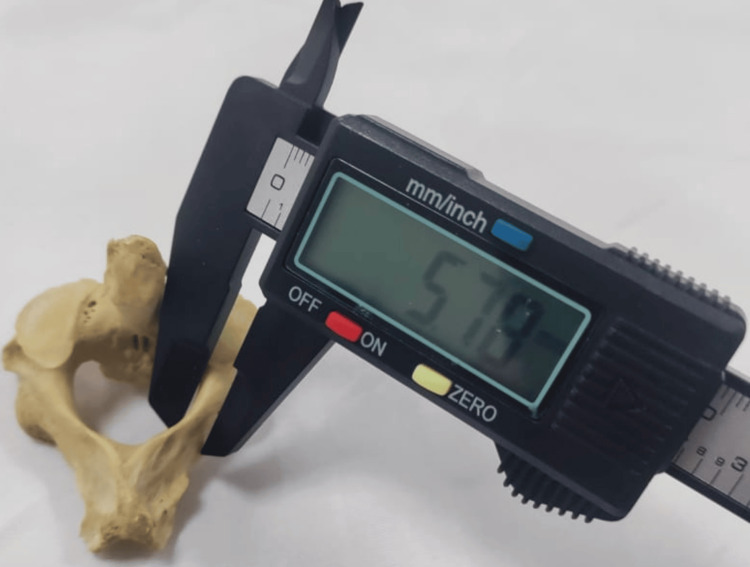
Measurement of the middle third of the laminar thickness of the axis vertebra Thickness was assessed at the upper, middle, and lower thirds of the lamina. The middle third represents the critical screw trajectory zone. The axis vertebra was sourced from Vardhman Mahavir Medical College and Safdarjung Hospital.

Thickness: It was assessed at the upper third, middle third, and lower third of the lamina. The middle third represents the critical screw trajectory zone (Figure 3).

Pedicle Measurements

Pedicle width: It was recorded as the maximum transverse diameter of the axis pedicle.

Statistical analysis

Data analysis was performed using the IBM SPSS Statistics software, version 21 (IBM Corp., Armonk, USA). A paired t-test was applied to compare morphometric measurements between the right and left sides. A p-value less than 0.05 was considered statistically significant. Descriptive statistics, including mean, standard deviation (SD), and range, were calculated and incorporated into the analysis. 

Risk stratification according to safety thresholds

Risk stratification of fixation corridors was performed for both the lamina and pedicle of C2 using a minimum safety threshold of ≥4.0 mm, as supported by previously published morphometric and surgical fixation studies [11-15]. Based on these criteria, fixation corridors were categorized into three risk groups. A safe corridor was defined as the laminar thickness or pedicle width measuring at least 4.0 mm bilaterally, permitting standard bilateral screw placement. A risky corridor was identified when the laminar thickness or pedicle width measured less than 4.0 mm on one side, necessitating modified screw trajectories or unilateral fixation. An unsafe corridor was defined as a laminar thickness or pedicle width of less than 4.0 mm bilaterally, representing an absolute contraindication to standard bilateral screw fixation.

## Results

Morphometry of the C2 lamina

The mean laminar length of the C2 vertebra measured 10.26 mm on the right side and 10.40 mm on the left side. Similarly, the observed mean height of the right lamina was 10.98 mm, and that of the left lamina was 10.84 mm. No significant statistical difference was found between the two sides with respect to height and length of the lamina of the axis vertebrae (Table 1).

**Table 1:** Morphometric observations of the C2 lamina SD: standard deviation; Min: minimum; Max: maximum; C2: second cervical vertebra

Parameters		Right	Left	P-value
Length (mm)	Range (Min-Max)	5.2-16.8	4.1-17	0.68
	Mean ± SD	10.26 ± 2.29	10.40 ± 2.26	
Height (mm)	Range (Min-Max)	7.6-14.3	6.3-14.5	0.75
	Mean ± SD	10.98 ± 1.57	10.84 ± 1.74	

Segmental thickness of the C2 lamina

No statistically significant side-to-side difference was observed in laminar thickness at the upper, middle, or lower thirds of the C2 lamina (p>0.05) (Table 2).

**Table 2:** Segmental thickness of the C2 lamina Middle laminar thickness represents the critical screw trajectory zone. SD: standard deviation; Min: minimum; Max: maximum; C2: second cervical vertebra

Parameters	Right (Mean ± SD)	Range Right (Min-Max)	Left (Mean ± SD)	Range Left (Min-Max)	P-value
Upper third (mm)	4.6 ± 0.9	3.4-6.8	4.5 ± 0.8	3.5-6.6	0.62
Middle third (mm)	5.8 ± 1.0	3.3-8.1	5.8 ± 1.1	3.3-8.0	0.91
Lower third (mm)	5.1 ± 0.9	3.6-7.4	5.0 ± 0.8	3.7-7.2	0.68

Notably, despite favorable mean thickness values, the observed minimum middle laminar thickness of 3.3 mm underscores the presence of clinically significant anatomical variability within the population.

Risk stratification based on the thickness of the middle third of the C2 lamina 

To translate morphometric findings into clinical applicability, specimens were stratified using a 4.0 mm safety threshold, which is commonly accepted as the minimum requirement for safe C2 TLS placement (Table 3).

**Table 3:** Risk stratification based on the thickness of the middle third of the C2 lamina (n=50) C2: second cervical vertebra

Thickness of the Middle Third of the Lamina	N	Percentage (%)
Bilateral safe laminae ≥4.0 mm	36	72.0
Unilateral unsafe lamina <4.0 mm	12	24.0
Bilateral unsafe laminae <4.0 mm	2	4.0

Bilateral laminar thickness ≥4.0 mm was observed in 36 (72%) vertebrae, indicating suitability for bilateral TLS fixation. In 12 (24%) vertebrae, one lamina measured below the safety threshold, suggesting increased procedural risk and modified trajectories or unilateral fixation. Bilateral laminar thickness less than 4.0 mm was identified in two (4%) specimens, rendering them unsuitable for TLS fixation. Overall, 14 (28%) axis vertebrae exhibited unilateral or bilateral laminar dimensions below the safety threshold, corresponding to the combined risky and unsafe categories.

Fixation corridor morphometry

Parameters directly determining screw corridor safety, namely middle third laminar thickness and pedicle width, showed substantial individual variability despite symmetrical mean values (Table 4).

**Table 4:** Fixation corridor morphometry SD: standard deviation; Min: minimum; Max: maximum

Parameters	Right (Mean ± SD)	Left (Mean ± SD)	Range Right (Min-Max)	Range Left (Min-Max)	P-value
Middle third lamina thickness (mm)	5.8 ± 1.2	5.8 ± 1.3	3.5-8.1	3.3-8.3	0.91
Pedicle width (mm)	5.19 ± 1.53	5.15 ± 1.79	2.1-10.5	2.2-10.5	0.91

No side-to-side significant statistical difference was observed in pedicle width and laminar thickness.

A critical observation was that the minimum laminar thickness was 3.3 mm and the minimum pedicle width was 2.1 mm, both falling below the accepted safety threshold of 4.0 mm, indicating an absolute anatomical risk in a subset of specimens.

Integrated risk stratification of C2 fixation corridors

Using a surgical threshold of ≥4.0 mm, specimens were stratified into safe, risky, and unsafe corridors for both TLS and TPS fixation (Table 5). 

**Table 5:** Integrated risk stratification of C2 fixation corridors (n=50) C2: second cervical vertebra

Corridor	Safe (Bilateral ≥4 mm)	Risky (One Side <4 mm)	Unsafe (Bilateral <4 mm)	Total
Translaminar screw	33 (66%)	13 (26%)	4 (8%)	50
Transpedicular screw	38 (76%)	10 (20%)	2 (4%)	50

For TLS fixation, 33 (66%) specimens demonstrated adequate thickness of lamina on both sides and were classified as safe, 13 (26%) specimens were risky due to unilateral inadequacy, and four (8%) specimens were bilaterally unsafe. For pedicle screw fixation, 38 (76%) specimens were safe, 10 (20%) were risky, and two (4%) were bilaterally unsafe. This integrated stratification demonstrates that a clinically relevant proportion of specimens fall into high-risk or unsafe categories despite favorable mean morphometric values.

## Discussion

A cervical spine injury is a potentially life-threatening condition that can result in severe and permanent neurological disability, making the choice of fixation critical [16]. Bilateral TLS fixation of the axis is described as a posterior stabilization technique that avoids vertebral artery injury by confining screw placement within the C2 lamina [11,12]. Despite this advantage, the primary structures at risk during screw fixation are the dura and spinal cord due to potential canal breach [11,17]. To mitigate this risk, Ma et al. [13] in their study proposed a modified technique in which the screw penetrates the dorsal cortex of the contralateral lateral mass, achieving bicortical purchase. These considerations underscore the clinical importance of precise morphometric evaluation of the C2 lamina.

The present study revealed average laminar length of 10.26 mm (right) and 10.40 mm (left) and average laminar height of 10.98 mm (right) and 10.84 mm (left), indicating symmetrical laminar morphology. Laminar dimensions reported by Xin-yu et al. [18] are smaller than the dimensions reported in the present study, suggesting possible population-based variation. The absence of bilateral asymmetry supports the feasibility of symmetric fixation strategies. However, absolute dimensions remain a critical determinant for screw safety.

Segmental analysis of laminar thickness showed variations across the upper, middle, and lower thirds. The middle third demonstrated the greatest mean thickness (5.8 mm bilaterally). No statistically significant side-to-side difference was observed at any level. Despite favorable mean values, the minimum thickness recorded was 3.3 mm, indicating substantial variations between individuals. This finding is clinically relevant as the middle third represents the preferred corridor for TLS placement. Using a widely accepted safety threshold of ≥4.0 mm, risk stratification revealed that 36 (72%) specimens were suitable for bilateral TLS fixation, 12 (24%) permitted unilateral or modified fixation, and two (4%) were bilaterally unsafe. Thus, 14 (28%) specimens exhibited at least one lamina below the safety threshold, emphasizing the risk of relying solely on mean morphometric values without individualized assessment. The present study also demonstrated side-wise variability, with unilateral mid-laminar thickness <4.0 mm, highlighting the need for individualized, side-specific evaluation, as fixation strategy may differ between right and left sides of the same C2 vertebra. 

Further evaluation of fixation corridor morphometry revealed symmetrical mean values of pedicle width, with no statistically significant side-to-side differences. Nevertheless, the minimum pedicle width recorded was 2.1 mm, well below the accepted safety threshold, indicating an absolute anatomical contraindication for pedicle screw placement in a subset of specimens. C2 lacks a distinct pedicle and instead forms a complex of an inferior pedicle and superior isthmus that is comparatively wider by about 3 mm [19]. Sengul and Kadiaglu [9] reported a mean C2 pedicle width of 9.5 mm, which is considerably larger than that observed in the present study, suggesting possible population-related differences. In another study [12], gender-based differences were observed, with a mean pedicle width of 5.0 ± 1.1 mm in male patients and 4.3 ± 1.1 mm in female patients. The present study revealed a mean pedicle width of 5.19 ± 1.53 mm on the right and 5.15 ± 1.79 mm on the left, comparable to the values reported in previous studies [12,19].

Previous CT-based studies have similarly demonstrated variability in C2 laminar dimensions [13,18-20]. Saetia and Phankhongsab [20] reported a mean inner transverse diameter of 4.23 ± 1.22 mm, with 79% of patients having dimensions suitable for TLS placement, and noted significantly larger diameters in male patients. Nakanishi et al. [21] reported smaller mean inner diameters (3.8 mm), possibly reflecting ethnic differences. Likewise, relatively smaller laminae were noted in a Malaysian population, and caution was advised when using 3.5 mm screws [15]. Many reports have demonstrated suitability rates ranging from 50% to 100% [13,14,22,23]. Ma et al. [13] noted that most of the specimens had bilaterally adequate laminar thickness, and a measurable proportion exhibited unilateral or bilateral dimensions below the safety threshold. These findings are in line with the present study and reinforce the need for individualized morphometric evaluation. 

Cadaveric and imaging studies support the feasibility of C2 TLS fixation [17-19,22,24]. However, observations on vertebral artery injury risk remain conflicting, with some studies documenting significant risk [25], while others report minimal or negligible risk with proper intraosseous placement [21,24,26]. No study has demonstrated an adequate diameter of the upper third of the C2 lamina for safe screw placement. Therefore, most authors strongly advocate preoperative CT evaluation, and in cases with marginal anatomy, three-dimensional reconstruction is recommended [22,27].

The present study highlights a significant proportion of anatomically unsafe corridors, although translaminar and pedicle screw fixation are feasible in the majority of specimens. The findings of the present study reinforce the necessity for individualized, specially side-wise preoperative imaging to enhance surgical safety and emphasize the limitations of relying on average morphometric values. Our study has several limitations, including a modest sample size and a single geographic region, which may limit generalization. Lack of demographic information hindered subgroup analysis, and the in vivo relationship with soft tissue and vascular structures could not be assessed. Future studies involving larger, multiregional samples and CT-based analysis may offer additional validation of these findings.

## Conclusions

The majority of C2 vertebrae in the present study demonstrated morphometric dimensions suitable for translaminar and pedicular screw fixation. However, a considerable proportion of specimens exhibited unilateral or bilateral dimensions below accepted safety thresholds, underscoring substantial individual and side-specific variability. These results show that relying on mean values alone is not enough, and meticulous preoperative CT evaluation is mandatory, with three-dimensional reconstruction in borderline cases. Larger, population-specific and imaging-based studies are needed to refine surgical planning and make C2 fixation techniques safer.
